# Ibuprofen supplementation and its effects on NF‐*κ*B activation in skeletal muscle following resistance exercise

**DOI:** 10.14814/phy2.12172

**Published:** 2014-10-24

**Authors:** Luke Vella, James F. Markworth, Jonathan M. Peake, Rod J. Snow, David Cameron‐Smith, Aaron P. Russell

**Affiliations:** 1 Centre for Physical Activity and Nutrition, School of Exercise and Nutrition Science, Deakin University, Burwood, Vic., Australia; 2 Liggins Institute, University of Auckland, Auckland, New Zealand; 3 School of Biomedical Sciences and Institute of Health and Biomedical Innovation, Queensland University of Technology, Brisbane, Qld, Australia

**Keywords:** Exercise recovery, inflammation, NF‐*κ*B, NSAID treatment, resistance exercise

## Abstract

Resistance exercise triggers a subclinical inflammatory response that plays a pivotal role in skeletal muscle regeneration. Nuclear factor‐*κ*B (NF‐*κ*B) is a stress signalling transcription factor that regulates acute and chronic states of inflammation. The classical NF‐*κ*B pathway regulates the early activation of post‐exercise inflammation; however there remains scope for this complex transcription factor to play a more detailed role in post‐exercise muscle recovery. Sixteen volunteers completed a bout of lower body resistance exercise with the ingestion of three 400 mg doses of ibuprofen or a placebo control. Muscle biopsy samples were obtained prior to exercise and at 0, 3 and 24 h post‐exercise and analysed for key markers of NF‐*κ*B activity. Phosphorylated p65 protein expression and p65 inflammatory target genes were elevated immediately post‐exercise independent of the two treatments. These changes did not translate to an increase in p65 DNA binding activity. NF‐*κ*B p50 protein expression and NF‐*κ*B p50 binding activity were lower than pre‐exercise at 0 and 3 h post‐exercise, but were elevated at 24 h post‐exercise. These findings provide novel evidence that two distinct NF‐*κ*B pathways are active in skeletal muscle after resistance exercise. The initial wave of activity involving p65 resembles the classical pathway and is associated with the onset of an acute inflammatory response. The second wave of NF‐*κ*B activity comprises the p50 subunit, which has been previously shown to resolve an acute inflammatory program. The current study showed no effect of the ibuprofen treatment on markers of the NF‐*κ*B pathway, however examination of the within group effects of the exercise protocol suggests that this pathway warrants further research.

## Abstract (precis)

The current study aimed to explore the regulation of the NF‐κB pathway following an acute bout of resistance exercise. Findings demonstrated two distinct phases of NF‐κB activity: an initial wave of activity comprising the p65 subunit, and a delayed second wave involving the p50 subunit.

## Introduction

Unaccustomed resistance exercise causes skeletal muscle damage that impairs muscle function and promotes sensations of pain. The precise signalling mechanisms that initiate muscle repair following exercise‐induced damage remain a topic of ongoing debate [previously reviewed by Paulsen et al. (2012)]. The onset of muscle damage triggers a complex interplay of intracellular events that involve myofibre injury, acute inflammation and cellular repair. At a symptomatic level, inflammation in skeletal muscle is characterised by swelling and soreness (Paulsen et al. 2012). Consequently post‐exercise inflammation is often ascribed as a cause of delayed‐onset muscle soreness (DOMS). DOMS typically occurs 24–48 h post‐exercise, and is accompanied by a secondary reduction in muscle force‐generating capacity. Treatment of DOMS has focused on reducing inflammation; however this may be detrimental to processes of cellular repair (Urso 2013). Attenuating exercise‐induced inflammation using non‐steroidal anti‐inflammatory drugs reduces skeletal muscle protein synthesis (Trappe et al. 2002) and impairs satellite cell proliferation (Mikkelsen et al. 2009). To develop more efficacious treatments for DOMS, a better understanding of the mechanisms that govern inflammation and tissue repair in skeletal muscle after exercise is required.

The nuclear factor‐kappa B (NF‐*κ*B) transcription factor acts as a central regulator of inflammatory signalling pathways. The NF‐*κ*B family consists of five subunits, including NF‐*κ*B1 (p105/p50), NF‐*κ*B2 (p100/p52), RelA (p65), RelB and cREL that share an amino terminus Rel homology domain. The Rel domain permits DNA binding, nuclear localization, dimerization and interaction with its own inhibitory protein I*κ*B (Delhalle et al. 2004; Mourkioti and Rosenthal 2008; Bakkar and Guttridge 2010). Under basal conditions RelA (p65), cREL and RelB subunits remain sequestered within the cytoplasm bound to an inhibitory I*κ*B protein. The I*κ*B family that regulates NF‐*κ*B includes the subunits I*κ*B*β*, I*κ*B*α*, I*κ*B*γ* I*κ*B*ε*, and Bcl‐3. Unlike the Rel proteins, subunits p50 and p52 are synthesized as large precursor proteins (p105 and 100, respectively) that require proteolytic processing to permit nuclear localization. These subunits lack a REL domain, to initiate gene transcription, and hence are primarily considered as repressors of gene transcription (Bonizzi and Karin 2004; Bakkar and Guttridge 2010).

Upon stimulation, the I*κ*B kinase complex (IKK) controls the degradation of I*κ*B and its precursor proteins, thereby enabling the NF‐*κ*B REL subunits to control gene transcription. The IKK complex consists of two catalytic kinases IKK*α* and IKK*β*, and a regulatory IKK*γ* subunit. IKK*β* activates the classical NF‐*κ*B signalling pathway through the phosphorylation and subsequent degradation of the inhibitor I*κ*B*α* (Zandi et al. 1997; Pahl 1999). The classical pathway typically comprises p65:p50 heterodimers, and is essential for the activation of acute inflammation by controlling the transcription on inflammatory cytokines and acute phase proteins (Zandi et al. 1997; Pahl 1999). More recently an alternative NF‐*κ*B pathway has been described, which is dependent on IKK*α* (Senftleben et al. 2001). Alternative signalling involves the activation of a secondary NF‐*κ*B inducing kinase (NIK), and has been linked to the activation of both p52 and p50 subunits. The functional significance of IKK*α*‐dependent gene expression in acute inflammation is not yet well established. However, preliminary research in rat muscle tissue suggests p50 homodimers may play a crucial role in the resolution of acute inflammation (Bohuslav et al. 1998; Lawrence et al. 2001).

Despite the importance of inflammation in tissue regeneration post‐exercise, very little is known about how NF‐*κ*B activity is regulated in skeletal muscle after acute exercise. A transient increase in various components of the classical NF‐*κ*B signalling pathway is observed in rat muscle post‐exercise (Hollander et al. 2001; Ji et al. 2004; Ho et al. 2005; Spangenburg et al. 2006; Kramer and Goodyear 2007). In contrast, a decrease in NF‐*κ*B DNA binding at 0 h post‐exercise with a return to near baseline 1 h post‐exercise was observed in human muscle following a traditional resistance exercise model (Durham et al. 2004). Recent work from our laboratory, using a similar resistance exercise model, demonstrated an increase in NF‐*κ*B binding to the promoter region of key inflammatory cytokines at 2 h post‐exercise, with a return to baseline levels at 4 h post‐exercise (Vella et al. 2012). These findings suggest that a transient NF‐*κ*B response contributes to acute post‐exercise inflammation.

To enhance our understanding of the cellular mechanisms that regulate both the onset and resolution of post‐exercise inflammation, the current study aimed to investigate changes in the activity of the subunits that comprise the classical and alternative NF‐*κ*B signalling pathways. We hypothesized that the regulation of NF‐*κ*B post‐exercise would involve two distinct waves of activation. Specifically, we hypothesized that the classical NF‐*κ*B pathway, involving p65 and p50 dimers would be activated soon after exercise during the early phases of inflammation, while the alternative pathway, comprising mainly the p50 subunit would be activated at later time points after exercise that correspond with the resolution of acute inflammation (Bohuslav et al. 1998; Ishikawa et al. 1998). We also hypothesized that the administration of ibuprofen would blunt both waves of NF‐*κ*B activation, providing a potential mechanism through which anti‐inflammatory medication might attenuate exercise‐induced inflammation in skeletal muscle.

## Methods

### Participants

As previously described, sixteen healthy male subjects were recruited to participate in the study (characteristics shown in Table 1) (Markworth et al. 2013). All participants completed a medical history questionnaire that was used to identify and exclude participants with a diagnosed condition or illness that prevented them from completing strenuous exercise. Exclusion criteria included participation in a lower body resistance exercise program within the last 6 months to ensure a muscle damage response from the exercise stimulus, and/or chronic treatment with anti‐inflammatory drugs. Current use of prescription medication or nutritional supplements also excluded subjects from participating.

**Table 1.** Subject characteristics and strength testing data.

	Characteristics				Strength (1RM)		
	Age (y)	Height (m)	Body mass (kg)	BMI	Squat(kg)	Leg press (kg)	Leg extension (kg)
PLA	23.9 ± 1.3	1.89 ± 0.1	86.9 ± 4.5	24.5 ± 1.2	94.9 ± 5.4	237 ± 17	236 ± 18
IBU	23.0 ± 0.5	1.89 ± 0.1	89.1 ± 4.4	24.8 ± 0.8	91.9 ± 6.0	240 ± 15	196 ± 22

Values are mean values ± SEM. No significant differences were observed between the two groups.

### Ethics approval

Prior to participation each subject received written and oral information regarding the nature of the experiment before providing written consent to participate. All procedures involved in this study were approved by the Deakin University Human Research Ethics Committee (DUHREC 2010‐019). All muscle sampling procedures were performed in accordance with the Helsinki declaration.

### Familiarization and strength testing

Each participant completed a familiarization session at least 7 days prior to completing the exercise trial. Participants performed repetition maximum testing to determine the experimental exercise load (80% of 1 repetition maximum (1RM)). The maximal weight each subject could lift was determined for the Smith machine‐assisted squat, the leg press, and the leg extension. These data were substituted into the validated Brzycki equation to predict 1RM for each participant (Mayhew et al. 1995; Whisenant et al. 2003). The participants were required to abstain from any further exercise until completion of the trial.

## Experimental Procedures

The participants reported to the laboratory in an over‐night fasted state, having abstained from caffeine, tobacco and alcohol for the preceding 24 h. Following 30 min of supine resting a pre‐exercise muscle biopsy was taken. Participants then completed a 10 min warm up consisting of low intensity cycling on a bicycle ergometer, and one low resistance set for each exercise at approximately 30–50% of the participants 1RM. Each participant then completed a single bout of intense resistance exercise. This session consisted of three sets of 8–10 repetitions of a bilateral Smith machine assisted squat, 45° leg press and leg extension. These exercises were all performed at 80% 1RM. The exercises were performed sequentially as a circuit, with 1 min rest between each exercise, and 3 min rest between sets. We have previously used this exercise protocol and demonstrated that it activates inflammatory signalling pathways (Vella et al. 2012). Following the completion of the exercise bout, the participants rested in a supine position while muscle biopsy samples were collected. The participants returned to the laboratory the following morning in an over‐night fasted state for a final muscle biopsy sample. Standardized meals were provided to participants the night preceding the trial (carbohydrate 57%, fat 22%, protein 21%), in the laboratory immediately following the exercise bout (carbohydrate 71%, fat 13%, protein 16%), as additional snacks throughout the day and an evening meal (carbohydrate 64%, fat 27%, protein 18%).

### NSAID administration

Prior to the exercise bout, the participants were randomly assigned to either the ibuprofen (NSAID) group (*n* = 8) or the placebo group (PLA) (*n *= 8). Participants in the NSAID group consumed the maximum recommended over‐the‐counter dose of 1200 mg of ibuprofen as three doses of 400 mg throughout the trial day. The first dose was administered upon arriving to the laboratory on the first morning of the trial, immediately prior to the first muscle biopsy sample. Participants were instructed to consume two additional doses at 2:00 pm and 8:00 pm the same evening. This dosing structure was prescribed to optimise levels of circulating ibuprofen to biologically active levels throughout the course of the trial day. This protocol has previously been validated by our research group in this same group of study participants and the same NSAID administration protocol (Markworth et al. 2013). Alternatively the placebo consumed a gelatin capsule containing powdered sugar, identical in appearance to the ibuprofen capsule.

### Sample collection

Muscle biopsy samples were obtained under local anaesthesia (Xylocaine 1%) from the vastus lateralis muscle of either leg using the percutaneous needle biopsy technique modified to include suction (Buford et al. 2009). Samples were obtained prior to exercise, immediately following exercise and at 3 and 24 h post‐exercise. The muscle biopsy sample obtained immediately post‐exercise was taken within 1–2 min of the completion of the exercise protocol, and will herein after be referred to as 0 h post‐exercise. The muscle biopsy procedure has been shown to trigger a local inflammatory response (Malm et al. 2000). To minimize interference from the biopsy procedure, samples prior to exercise and at 24 h post‐exercise were taken from the same leg, while muscle samples obtained at 0 and 3 h post‐exercise were taken from the opposite leg. Samples within the same leg were taken at least 5 cm from the previous site. We have previously reported that this technique is effective for minimizing cytokine gene expression and NF‐*κ*B activity in response to muscle biopsies (Vella et al. 2012). Excised tissue was immediately immersed in liquid nitrogen and stored at −80°C until further analysis.

### Subjective assessment of DOMS and range of movement

Subjective assessment of muscle soreness (DOMS) and range of movement were recorded prior to exercise and at 24 h post exercise. Subjects were asked to rate their levels of muscle soreness and range of movement on a 0–10 scale. In both instances 0 was considered as the best possible result and a rating of 10 was considered to be the worst result.

### Protein extraction and quantification

Muscle samples were homogenized in ice cold RIPA buffer (50 mmol/L Tris‐HCl, pH 7.4, 150 mmol/L NaCl, 0.25% deoxycholic acid, 1% NP‐40, 1 mmol/L EDTA supplemented with protease and phosphatase inhibitors including 1 mmol/L PMSF, 1 *μ*g/mL aprotinin, 1 *μ*g/mL leupeptin, 1 mmol/L Na_3_VO_4_ & 1 mmol/L NaF). The homogenate was agitated for 1 h at 4°C and centrifuged at 13,000 × *g* at 4°C for 15 min. The resultant supernatant was removed and stored at −80°C until further analysis. Total protein concentration was determined using a BCA protein assay kit according to the manufacturer's instruction (Pierce, Rockford, IL). Protein samples (50 *μ*g) were denatured in loading buffer and separated by a 10% SDS‐PAGE and transferred to a PVDF membrane. Membranes were blocked for 90 min at room temperature in 5% BSA/Tris buffered saline with 0.1% Tween 20 (TBST). Primary antibodies [phosphorylated NF‐*κ*B p65 Ser^536^, total NF‐*κ*B p65, NF‐*κ*B p100/p52, NF‐*κ*B p105/p50, NF‐*κ*B cREL and *β*‐actin (all obtained from Cell Signaling Technologies, Arundel, QLD)] were diluted to 1:1000 in 5% BSA/TBST, applied and incubated overnight at 4°C with gentle agitation. Membranes were washed for 30 min in TBST and probed with HRP conjugated secondary antibodies diluted to 1:2000 in 5%BSA/TBST, and incubated for 1 h at room temperature. Proteins were visualised by using Western Lighting enhanced chemiluminescence (Perkin Elmer Lifesciences, Boston, MA). Signals were captured using a Kodak Digital Image Station 2000M (model: 440CF; Eastman Kodak, Rochester, NY) and quantified by densitometry band analysis using Kodak Molecular Imaging Software (Version 4.0.5, © 1994–2005, Eastman Kodak). Phosphorylated NF‐*κ*B p65 protein was normalized to total p65 protein (Fig. 2A). NF‐*κ*B p50 protein expression was normalized to its precursor protein p105 (Fig. 2B). NF‐*κ*B p52 and cREL protein was normalized to *β*‐actin (Fig. 2C).

### RNA extraction and RT‐PCR

Total cellular RNA was extracted as previously described (Trenerry et al. 2007) using the ToTALLY RNA Kit (Ambion, Austin, TX). RNA quality and concentration were determined using the Agilent 2100 Biolanalyzer (Agilent Technologies, Palo Alto, CA). First strand cDNA was generated from 0.5 *μ*g total RNA using the AMV RT kit (Promega, Madison, WI). RT‐PCR was performed in duplicate using the Biorad CFX384 system (Biorad, Hercules, CA), containing 5XHOT FirePol^®^ EvaGreen Mix (Integrated Science, Sydney, NSW), forward primer, reverse primer, sterile nuclease free water and cDNA (0.125 ng/*μ*L). Data were analysed using a comparative critical threshold (Ct) method, where the amount of the specified target gene normalised to the amount of endogenous control, relative to the control value is given by 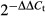 . The endogenous control used in this experiment was GAPDH. The efficacy of GAPDH as an endogenous control was examined using the equation 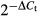 . Primers were designed using Primer Express software package version 3.0 (Applied Biosystems, Mulgrave, Vic., Australia). Gene sequences were obtained from GenBank (Table 2). Primer sequence specificity was confirmed using BLAST. A melting point dissociation curve was generated by the PCR instrument for all PCR products to confirm the presence of a single amplified product.

**Table 2.** Primer sequences were designed using Primer Express Software v 3.0 (Applied Biosystems) using sequences accessed through Genbank and checked for specificity using nucleotide‐nucleotide BLAST search.

Gene	Accession No.	Primer sequence
GAPDH	NM_21130	Forward: CAT CCA TGA CAA CTT TGG TAT CGT
		Reverse: CAG TCT TCT GGG TGG CAG TGA
MCP‐1	NM_002982	Forward: TCC CAA AGA AGC TGT GAT CTT CA
		Reverse: CAG ATT CTT GGG TGG AGT GA
IL‐6	NM_000600	Forward: GCG AAA GGA TGA AAG TGA CCA T
		Reverse: AGA CAA GCC CAG CAA TGA AAA
IL‐8	NM_000584	Forward: CTG GCC GTC GCT CTC TGG
		Reverse: TTA GCA CTC CTT GGC AAA ACT
TNF‐α	X_02910	Forward: GGA GAA GGG TGA CCG ACT CA
		Reverse: TGC CCA GAC TCG GCA AAG
COX‐2	U_20548	Forward: GAA TCA TTC ACC AGG CAA ATT G
		Reverse: TGG AAG CCT GTG ATA CTT TCT GTA CT

### Nuclear extraction and Transcription Factor (TF) Assay

Nuclear and cytoplasmic proteins were extracted from 20 mg of muscle tissue using a NE‐PER Nuclear and Cytoplasmic Extractions Reagents (Pierce, Rockford, IL), according to the manufacturer's instructions. Western blot analysis probed for GAPDH, *α*‐tubulin and Lamin A was performed to ensure the nuclear extract was not contaminated by cytoplasmic proteins. A Transcription Factor Assay detecting specific transcription factor DNA binding activity was performed according to manufacturer's instructions (Cayman Chemical, Ann Arbour, MI). Briefly, 10 *μ*g of nuclear protein were loaded into a well containing an immobilized NF‐*κ*B consensus sequence (5′GGGACTTTCC‐3′) and incubated overnight at 4°C. Primary antibodies for NF‐*κ*B subunits p65 and p50 were loaded in to each well and incubated at room temperature for 1 h. Each well was flushed using a diluted wash buffer for 30 min. Following a secondary 30 min wash, samples were incubated with secondary HRP‐conjugated antibody for a further 1 h at room temperature. To quantify transcription factor binding, a developing solution containing a 3,3′,5,5′‐Tetramethhylbenzidine (TMB) solution was added to each well and incubated for 45 min at room temperature. DNA binding was then quantified using photospectrometry with absorbance measurements taken at 450 nm using a Multiscan RC plate reader (Labsystems, Finland) and Gen5 Data Analysis Software (BioTek, Winooski, VT).

### Statistics

Data are expressed as means ± SEM. Prior to analysis the data displaying a lack of normality were log transformed to stabilise variance. Data were analysed using a two‐way ANOVA with repeated measures for time. The sphericity adjustment was checked, and if required, a Greenhouse‐Geisser epsilon to the residual degrees of freedom was applied. A data point was considered to be a statistical outlier where a z‐score exceeded a pre‐determined threshold of ± 4.00. Where appropriate we explored within‐group pair‐wise comparisons between individual time‐points using the Least Significant Differences of means at *P *< 0.05 to determine statistically significant changes (Mead 1988; Saville 1990). Statistical analyses were performed using GenStat for Windows 16th Edition (VSN International, Hemel Hempstead, UK).

## Results

Subjective DOMS analysis showed a main effect for time (time effect *P* < 0.01) with no effect for treatment. The average post‐exercise DOMS score was 4.81 ± 0.5 and the post‐exercise ROM score was 3.3 ± 0.5. This data demonstrates that the exercise protocol induced a moderate level of muscle soreness and this effect was irrespective of ibuprofen treatment.

**Figure 1.**
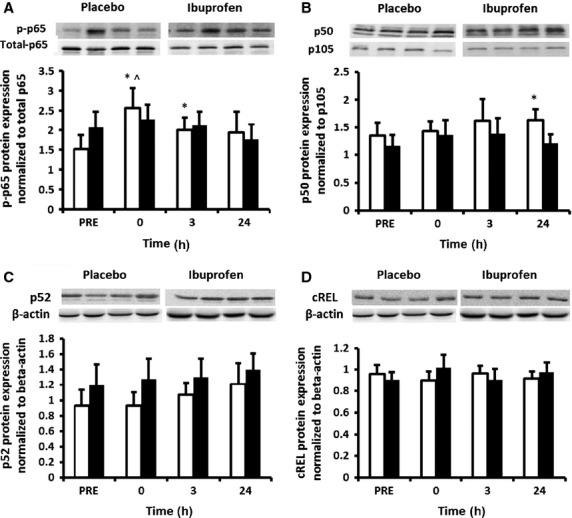
Protein expression of NF‐*κ*B subunits p65, p50, p52 and cREL. Representative Western blots for p‐p65 normalized to total p65 (A), p50 normalized to p105 (B), p52 normalized to *β*‐actin (C) and cREL normalized to *β*‐actin (D) measured in muscle biopsy samples. Data are mean arbitrary units ± SEM. *denotes statistical significance from pre exercise values in the placebo group; ^denotes statistical significance from 24 h post‐exercise in the control group (*P* < 0.05). White bars = placebo group; black bars = ibuprofen group.

**Figure 2.**
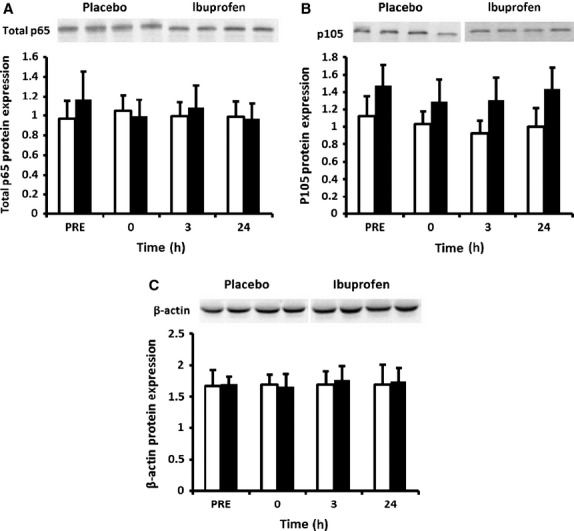
Protein expression of total NF‐*κ*B p65, NF‐*κ*B p105 and *β*‐actin. Data are mean arbitrary units ± SEM. White bars = placebo group; black bars = ibuprofen group.

Expression of NF‐*κ*B phosphorylated p65 (Ser 536) increased over time (time effect *P* = 0.006), but this response was overall not different between the groups (interaction effect *P* = 0.253, treatment effect *P* = 0.176). Nevertheless, LSD pairwise comparisons indicated that the main increase from baseline was in the placebo group at 0 h post‐exercise. Within the placebo group phosphorylated p65 protein expression remained elevated at 3 h post‐exercise and returned to baseline by 24 h (Fig. 1A). No significant changes were observed in the ibuprofen group by LSD comparisons. There was no effect of time or treatment for NF‐*κ*B total p65 (Fig. 2A).

There was a trend toward a significant time × treatment interaction (*P* = 0.057) for the protein expression of the p50 subunit, although a main effect for time (*P* = 0.376) or treatment (*P* = 0.865) was not achieved. Our analysis revealed a statistical outlier in the ibuprofen group at the 24 h time point. When this subject was removed from the analysis, this trend was weaker (*P* = 0.115) (Fig. 1B). LSD comparisons indicated an increase in p50 protein expression was observed at 24 h post exercise in the placebo group only (Fig. 1B). There were no main or interaction effects for the NF‐*κ*B p50 precursor protein p105 (Fig. 2B) or for p52 and cREL subunits (Fig. 1C and D).

**Figure 3.**
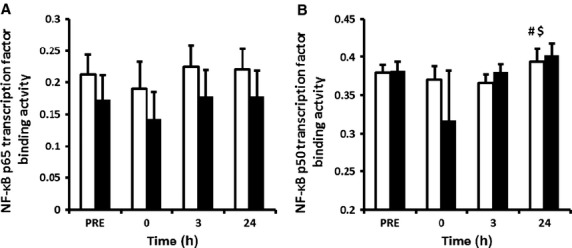
NF‐*κ*B subunits p65 (A) and p50 (B) binding to nuclear protein. Data are mean arbitrary units ± SEM. ^#^denotes statistical significance from 0 h post‐exercise in the control group; ^$^denotes statistical significance from 3 h post‐exercise in the control group. White bars = placebo group; black bars = ibuprofen group.

To determine subunit‐specific DNA binding of NF‐*κ*B following exercise, transcription factor binding assays were performed for p50 and p65 subunits. NF‐*κ*B p50 showed a main effect for time (*P* = 0.035), with no time × treatment interaction (*P* = 0.851) or main effect for treatment (*P* = 0.488) (Fig. 3B). LSD comparisons identified a significant increase in p50 binding that occurred at 24 h post‐exercise when compared to 0 and 3 h post‐exercise within the placebo group. This coincided with the increase in p50 protein expression (Fig. 1B). No change in p65 DNA binding was observed, despite an increase in phosphorylated p65 protein expression (Fig. 3B).

**Figure 4.**
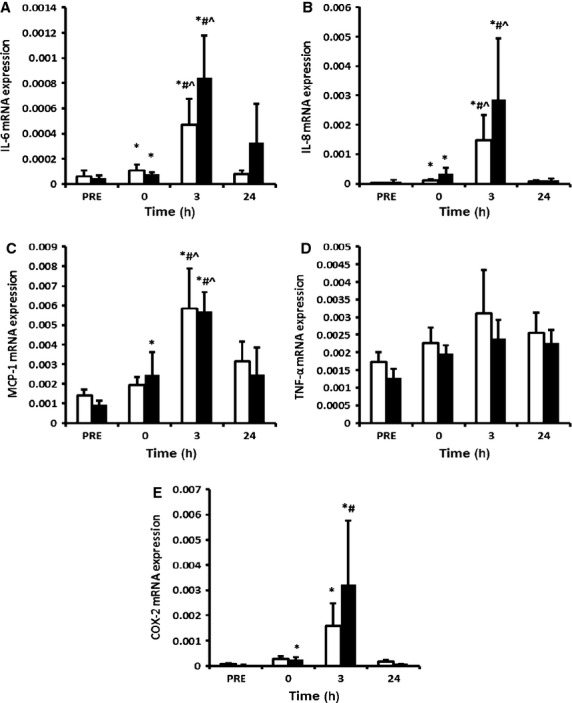
RT‐PCR analysis of NF‐*κ*B target genes IL‐6 (A), IL‐8 (B), MCP‐1 (C), TNF‐*α* (D) and COX‐2 (E) in skeletal muscle cDNA. Data are mean arbitrary units ± SEM. *denotes statistical significance from pre exercise in the same treatment group; ^#^denotes statistical significance from 0 h post‐exercise in the same treatment group; ^denotes statistical significance from 24 h post‐exercise in the same treatment group (*P* < 0.05). White bars = placebo group; black bars = ibuprofen group.

We sought to determine whether any increase in NF‐*κ*B signalling coincided with an increase in downstream inflammatory cytokines, and if the administration of ibuprofen influenced post‐exercise cytokine expression. The mRNA levels of IL‐6 (Fig. 4A), IL‐8 (Fig. 4B) and MCP‐1 (Fig. 4C) demonstrated a main effect for time (*P* < 0.01), with the highest elevation in expression levels at 3 h post‐exercise after significant increases at 0 h post‐exercise. TNF‐*α* mRNA remained unchanged after exercise (Fig. 4D). COX‐2 mRNA showed a main effect for time (*P* < 0.01), increasing at 0, 3 and 24 h post‐exercise (Fig. 4E). There were no significant interaction effects or main effects for treatment for the inflammatory cytokines or COX‐2 mRNA expression. Consistently, LSD pairwise comparisons revealed similar changes over time from baseline within both groups.

## Discussion

The current study aimed to explore the regulation of the different NF‐*κ*B subunits following a single bout of lower body resistance exercise, and investigate the mechanism through which ibuprofen treatment may influence post‐exercise inflammation. The results of this study show for the first time that an alternative NF‐*κ*B signalling pathway comprising mainly p50 subunits is activated 24 h post‐exercise. This pathway appears distinctly different to the activation of NF‐*κ*B p65 subunit that occurs during the early stages of acute post‐exercise inflammation.

Phosphorylated NF‐*κ*B p65 protein expression was significantly elevated in the placebo group when measured at 0 and 3 h post‐exercise, and returned to baseline levels at 24 h post‐exercise. These findings support those of previous research showing a post‐exercise increase in key components of the classical NF‐*κ*B signalling pathway in skeletal muscle, including phosphorylated NF‐*κ*B p65 protein (Ji et al. 2004; Vella et al. 2012), phosphorylated I*κ*B*α* protein (Ho et al. 2005; Vella et al. 2012) and NF‐*κ*B p65 DNA binding activity (Tantiwong et al. 2010; Hyldahl et al. 2011). In the present study, the increase in phosphorylated NF‐*κ*B p65 protein expression was not associated with an increase in p65 transcription factor binding activity at 0, 3 and 24 h post‐exercise. However, NF‐*κ*B regulated cytokine genes including MCP‐1, IL‐6 and IL‐8 were increased at 3 h post‐exercise. Previous work from our group showed an increase in NF‐*κ*B p65 binding to the promoter region of genes coding for inflammatory cytokines at 2 h after traditional resistance exercise (Vella et al. 2012). This research performed a series of electrophoretic mobility shift assays that looked specifically NF‐*κ*B binding to genes coding MCP‐1, IL‐6 and IL‐8 and thus differences in the methodology may explain conflicting results. Furthermore, the discrepancy in the results between our two trials may be explained by the short half‐life of NF‐*κ*B in the absence of an activating stimulus. In a HL60 cell line, the half‐life of NF‐*κ*B has been reported to be less than 30 min (Hohmann et al. 1991). Likewise, the inhibitory I*κ*B*α* protein has a half‐life of 25 min in Jurkat cells (Dodd et al. 2009). Therefore, in the present study, NF‐*κ*B activation may have returned to resting levels by 3 h post‐exercise. Research models using an extreme eccentric resistance exercise model were able to demonstrate an increase in p65 DNA binding to nuclear protein at 3 h post‐exercise (Hyldahl et al. 2011; Xin et al. 2013). By contrast, research from Durham et al. (2004) showed a decrease in NF‐*κ*B DNA binding activity immediately post‐exercise, which returned to baseline levels at 1 h post‐exercise. Collectively, these findings suggest that the activation of NF‐*κ*B DNA binding occurs transiently within the first few hours of exercise. Changes in NF‐*κ*B activity in skeletal muscle may depend on the mode of exercise and training status of participants.

This research also provides novel evidence for a potential role of NF‐*κ*B p50 activation as a component of inflammation‐resolution. In our initial analysis of p50 protein expression, there was a trend towards a time × group interaction effect (*P* = 0.057) but this trend was weaker (*P* = 0.115) after removing a statistical outlier. Despite this weaker interaction, within‐group analysis by LSD comparisons revealed that p50 protein expression was elevated in the placebo group at 24 h post‐exercise. Coincident with this response, p50 transcription factor binding activity was highest at 24 h post‐exercise. The biological significance of this delayed increase in NF‐*κ*B p50 signalling during the latter stages of post‐exercise recovery remains uncertain. However, *in vitro* work suggests that it may play a role in regulating the active resolution of acute inflammation in skeletal muscle (Lawrence et al. 2001, 2005; Senftleben et al. 2001). In a model of carageenin‐induced pleurisy in rats, Lawrence et al. (2001) provided preliminary evidence for a complex interplay between distinct NF‐*κ*B signalling pathways that control an acute and transient inflammatory response. They reported that the preliminary phase of NF‐*κ*B activation that occurred at 6 h was characteristic of the classical NF‐*κ*B pathway, and was associated with the onset of acute inflammation. The secondary phase was typical of the alternative NF‐*κ*B pathway, comprising p50 homodimers. Importantly, inhibiting this wave of activity caused a prolonged inflammatory response (Lawrence et al. 2001). Findings from the present study support the concept of two functionally distinct waves of NF‐*κ*B activity following traditional resistance exercise. Future work is warranted to determine how this secondary wave of NF‐*κ*B pathway activity influences post‐exercise inflammation and skeletal muscle recovery.

We used ibuprofen supplementation to investigate whether manipulating the inflammatory response to exercise through the cyclooxygenase‐prostaglandin pathway alters post‐exercise NF‐*κ*B signalling. Previous reports indicate that NF‐*κ*B can function upstream of COX‐2 to control transcription of this gene (Pahl 1999; Poligone and Baldwin 2001). Alternatively, prostaglandin activity may also affect NF‐*κ*B (Rossi et al. 2000; Straus et al. 2000; Poligone and Baldwin 2001). *In‐vitro* studies demonstrate that the effect of prostaglandins on NF‐*κ*B activity is specific to the class of prostaglandin. Prostaglandin E_2_ activates the classical NF‐*κ*B pathway, whereas prostaglandin A_2_, and prostaglandin J_2_ and its downstream analogues can inhibit NF‐*κ*B activation in response to pro‐inflammatory stimuli (Castrillo et al. 2000; Rossi et al. 2000; Straus et al. 2000; Lawrence et al. 2002). Our group recently reported changes in prostaglandins in blood serum samples after traditional resistance exercise (Markworth et al. 2013). Interestingly, PGE_2_, PGA_2_ and PGD_2_ all peaked in expression at 2 h post‐exercise. We did not find any significant time × group interaction effects for the change in p65 phosphorylation; however, LSD comparisons suggested that phosphorylated p65 expression only seemed to increase in the placebo group. Similarly the observed changes in p50 protein expression and DNA binding were only identified within the placebo group. These findings are supported by data from the study by Lawrence et al. (2001), which demonstrated no change in the secondary wave of NF‐*κ*B DNA binding activity following the administration of an alternative COX inhibitor, NS398, in rats with pleurisy. While these findings do not offer any conclusive evidence that ibuprofen treatment inhibits the NF‐*κ*B pathway, it does provide justification to further explore this pathway as a potential mechanism through which ibuprofen treatment inhibits post‐exercise inflammation.

There were several limitations to the present study that need to be considered. Firstly, this analysis was run as part of a larger study investigating the effects of ibuprofen administration on multiple components of the post‐exercise inflammatory and hypertrophy response (Markworth et al. 2013, 2014). Therefore, limited muscle sample remained to complete further analyses. Future research should consider the post‐exercise regulation of upstream markers including IKK*α* and IKK*β*.

## Conclusion

This research provides new insights into the regulation of NF‐*κ*B following a bout of acute resistance exercise. The primary finding from this study is that NF‐*κ*B activation follows a biphasic activation pattern that is subunit‐specific. The first wave involves the classical NF‐*κ*B p65 subunit, and corresponds with the onset of an acute inflammatory response and an increase in inflammatory cytokine gene expression. The second wave is consistent with a previously identified secondary wave of NF‐*κ*B activity that involves the alternative p50 subunit. Research in alternative models of inflammation suggests that this second wave of activity may be associated with an active inflammatory resolution program. Further research into the role of p50 signalling in skeletal muscle represents a key area for future research in order to better understand the mechanisms that regulate post‐exercise inflammation. Analysis of the LSD to examine pair‐wise within‐group differences suggested that the observed changes in both NF‐*κ*B p65 and p50 were detected only within the placebo group. The complex interplay between the NF‐*κ*B and the COX/prostaglandin pathway in exercise‐induced muscle damage remains poorly understood, and should be a focus for future research.

## Acknowledgments

The authors would like to acknowledge to assistance of Associate Professor John Reynolds for his contributions in the statistical analysis of the dataset.

## Conflict of Interest

There were no conflicts of interest relevant to this paper.
